# Peer review of "A Single-Cell Perspective on the Effects of Dopamine in the Regulation of HIV Latency Phenotypes in a Myeloid Cell Model"

_viruses, 2025, doi:10.3390/v17070895_

Round 1

Reviewer 1 Report (Previous Reviewer 1)

Comments and Suggestions for Authors

The authors have provided detailed explanations for my comments. Although I would have liked to see some additional IHC studies on differentially expressed latency associated genes in brain samples of SIV infected macaques on ART and methamphetamine, I am okay with the authors plan to do these studies in the future.

Author Response

Thank you so much for all the comments. We are working in the experiments using macaques and soon we will have exciting news.

Reviewer 2 Report (Previous Reviewer 2)

Comments and Suggestions for Authors

In the revised manuscript, the authors have responded to the comments in the previous version and have added additional Figures to support their findings. The authors show the results of p24 released in the culture medium from not treated, 1, µM, 10 µM, and iBET treated cells. These results indicate that significant levels of p24 are released from the not treated group.  Thus,  I still have concerns regarding its relevance and I believe that results from primary cells should be included. 

Reviewer comment #1: Viral latency generally refers to a state where a virus remains dormant within an infected host cell, neither actively replicating nor causing immediate symptoms, but capable of reactivating and resuming the infectious cycle under specific conditions. In this manuscript, the authors examine the role of dopamine (10µM) on host gene expression and latency reversal in the parental U937 cell line and U1 cells, a derivative of U937 cells with two integrated copies of the NY5 strain of HIV-1. The single-cell transcriptomics is well described with adequate controls and analyzed with relevant software. However, there are several concerns regarding this study. The first concern is that the reader is left with numerous either up-regulated or down-regulated genes and no experiments showing they are involved in latency reversal.

Author’s Response: Thank you so much for this comment. We agree with the reviewer that there is a lot going on, and we provided a visual summary with the main signatures by condition and what we believe is the role they are playing. But in fact, this strategy has generated data that allow us to make predictions. Currently we are focused on the DA-induced decrease in H1 histone linkers (which I added a sample to supplementary materials) that bind at the nucleosome entry and exit sites in a dynamic manner to form higher-order chromatin structures. Those findings can be considered a validation, but are currently being organized in the format of another manuscript that will stress specifically these predictions. For example, H1.5 shows a decrease caused by DA in a dose-dependent manner at the protein level, visualized in nuclear extracts in an inverse correlation with open chromatin markers such as H3K36me3, and increase in p24. This effect seems to match a decrease in Sirtuin-1 binding (not shown but will be in the new paper), which plays a role in H1 stabilization with transcriptional silencing as a result. Most important, we already know that the effect is replicated in U1 cells, latently infected C20 microglia, and infected primary microglia (study in progress). We have opted to have this present manuscript presented mainly as a method to distinguish phenotypes associated with positive HIV transcription using single cell approaches, and later show the effects in predicted epigenetic marks as a robust separate sequelae paper.

New Reviewer comments:   First, I do believe that the U1 cell line is cloned. The abstract from the Folks et al. paper states, “The parent promonocyte cell line U937 was chronically infected with HIV-1 and from this line a clone, U1, was derived.”  A major concern is the p24 results (Figure S1). On the graph presented, the NT (I assume not treated) showed nearly 1000ng/ml of p24 in the culture supernatant, while the 1 µM DA resulted in ~1200ng/mL, and 10 µM DA resulted in ~ 8000ng/mL.  In my years of performing p24 assays, I’ve never had a control (in this case the Not Treated control) of nearly 1000 ng/mL; it should rather be ~20-50 pg/mL).  From these results, one can conclude that U1 cell line is not latently infected but rather is virus-producing.  The authors should include the data from latently infected C20 microglia (an SV40 T Agn, hTERT immortalized cell line) and infected primary microglia. This would provide more a more comprehensive comparison.

Reviewer comment #2:  The second concern is using the U1 cell line, a derivative of the U937 cell line first isolated from an individual with adult acute monocytic leukemia. In a previous study, investigators investigated the integration sites in several “latently infected” cell lines, including the U1 cell line. They showed that HIV-1 was mainly integrated into chromosome 2 and the X chromosome, with less frequent integration into chromosome 19. Based on sequence analysis, these investigators found a total of 28 unique HIV integration sites, indicating that these cell lines are not clonal (Symons et al., Retrovirology, 14:2, 2017). The above study suggests that the results of the transcriptomics studies could vary based on the number of cell populations with different HIV-1 integration sites.

Author’s Response: The reviewer is correct, and this is a brilliant comment that could explain the heterogeneity in the response to DA, which alone indicates that the model is not clonal. In the context of myeloid cell targets, clonality is probably not as relevant as it is the context of T cells with features driven by specificity. But the baseline production of virus that can integrate in multiple sites is a factor that eliminates the concept of clonality from the picture. We do not recall using the word “clonality”, but the heterogeneity, especially at baseline, is however a strength, because in the brain, myeloid cells are highly heterogeneous in phenotypes and responses, especially microglia subsets across regions of the brain. On the other hand, U937 cells also showed signs of heterogeneity, both at baseline and dynamically in response to DA, indicating that other factors could cause a diversity in the response regardless of integration. We have cited the recommended works and papers by different groups, using other cell lines and describing a similar phenomenon of heterogeneity regardless of viral infection. Yet, we agree that integration numbers and sites are important. As mentioned by the reviewer, identifying the specific integration sites in U1 cells has been previously addressed in the literature (now added to the manuscript with improved citations – thank you) and is beyond the scope of this work. Other approaches would be necessary to establish that at the single cell level and in respect with the transcriptome. However, such study is ongoing in our lab, in latently HIV-infected microglia cells (which is suggested by this reviewer below), using both cell lines and primary cells. Thus, we are applying this developed pipeline to other models, and we are adding upon the findings reported here, to validate the mechanistic insights in more brain-relevant models and in respect to mapped integration sites. We will soon have a new manuscript. In the meantime, we have added a piece of discussion to the revised version of the manuscript on the potential impact of integration site numbers and location to the development of phenotypes and response patterns, and we have included the suggested reference as well as other (line 629).

New Reviewer Comment:  First, see my comment above regarding the p24. The use of primary cell types such as microglia and/or monocyte derived macrophages (MDM) is essential as they represent a more physiological cell type. 

  1. Reviewer comment: The third concern is whether the data from these transcriptional studies can be related to myeloid cells from HIV-1-positive individuals without leukemia. Better models have been described for assessing HIV-1 latency in primary monocyte-derived macrophages (MDM) (Wong et al., J. Virology; 95:(19):e0022721, 2021).

Author’s response: This is a fascinating question. Indeed, U937 cells, modified to express integrated virus (U1), are derived from a leukemia patient. For this reason, the overlap and comparison between uninfected, untreated, and infected and treated is very important. In the overlapping features, we have identified tumoral pathways, but did not turn out significant upon the comparison between DA-stimulation and infected conditions and were excluded from the visualizations by the analysis software calibrated with setting thresholds designed to identify unique cluster characteristics. Regarding the use of MDMs, we have collaborators applying the approach described in this manuscript to detect patient characteristics and phenotypes. The approach we developed and presented here as a method can be used in MDMs to characterize clinical cases in HIV+ populations with issues of substance use. The variability between subjects, though, needs to be considered to achieve the necessary power. However, this is an important point, which we are mindful about, and currently working with collaborators to analyze cluster responses in an individual basis using MDM as a powerful strategy.  We very much appreciate the comment and the suggestion.

Reviewer’s response:  The authors suggest that analysis of patient MDMs may lead to  variability between subjects. It would be to show this with primary MDM cells, if in fact they are latently infected. This also further justifies why the inclusion of primary MDM should be included in this studied.

  1. Reviewer comment: A fourth concern relates to the expression of HIV-1. The U1 cell line has two integrated proviruses, both of which have mutations in tat (in one integrant the tat initiation codon is mutated to a leucine resulting in no Tat produced; in the second integrant, there is an amino acid substitution (H to L) at position 13, resulting in significantly decreased transactivation activity and virus production (see Emiliani et al., J. Virology, 72:1666-1670,1998). While the authors show HIV-1 transcripts in their studies, this should be confirmed with studies measuring the levels of viral proteins released (either by p24 or reverse transcriptase assays) following “latency reversal” by dopamine. Such studies need to be included to support their data. Finally, the manuscript would be strengthened by a) assessing transcripts in the U1 cell line treated with phorbol 12-myristate 13-acetate (PMA) or other known latency reversal agents and comparing them with those of the dopamine studies; and b) assessing transcripts in the U1 cell line treated with a dopamine antagonist before dopamine treatment.

Author’s response: Thank you very much for this comment, which is shared by the previous reviewer. We have added the results of p24 levels in the culture supernatants, measured specifically for this experiment, but also highlighted in the discussion that this has been previously published by us. The figure shows the effects of DA on p24 and the comparison with a latency reversal agent (iBet151) can be appreciated.

Reviewer’s response: A major concern is the p24 results (Figure S1). On the graph presented, the NT (I assume not treated) showed nearly 1000ng/ml of p24 in the culture supernatant, while the 1 µM DA resulted in ~1200ng/mL, and 10 µM DA resulted in ~ 8000ng/mL.  In all of my years of performing p24 assays, I’ve never had a control of nearly 1000 ng/mL. This experiment shows that the U1 cell line is not latently infected. Finally, why didn’t the authors test DA concentrations of U937 cells?

Author Response

I would like to again thank the reviewers for their great insights and comments, and the exciting discussion. Please, find below a point-by-point response to all remaining issues.

  • Regarding whether U1 cells are a clone and a model of latency.

New Reviewer comments:   First, I do believe that the U1 cell line is cloned. The abstract from the Folks et al. paper states, “The parent promonocyte cell line U937 was chronically infected with HIV-1 and from this line a clone, U1, was derived.”  A major concern is the p24 results (Figure S1). On the graph presented, the NT (I assume not treated) showed nearly 1000ng/ml of p24 in the culture supernatant, while the 1 µM DA resulted in ~1200ng/mL, and 10 µM DA resulted in ~ 8000ng/mL.  In my years of performing p24 assays, I’ve never had a control (in this case the Not Treated control) of nearly 1000 ng/mL; it should rather be ~20-50 pg/mL).  From these results, one can conclude that U1 cell line is not latently infected but rather is virus-producing.  The authors should include the data from latently infected C20 microglia (an SV40 T Agn, hTERT immortalized cell line) and infected primary microglia. This would provide more a more comprehensive comparison.

Response: Dear Reviewer, you are right about the levels of p24. We double checked the methods in the kit, and the values are in pg/ml, not ng/ml. I am very grateful for your attention in this matter, and we corrected the figure. We do not have experiments in MDMs, but we do have latency experiments in C20s and in primary microglia using HIV GKO. The experiments in microglia systems are intended to another work, but I provided the p24 levels in graphs, side by side, for your appreciation, in supplementary materials. I want to highlight that the results in C20 cells are in ng/ml, and that the effect of DA is replicated in all systems. Similar to U1s, C20s are cell lines, constantly proliferating, which may account to a higher baseline. Yet, despite this limitation, and again the fact that there are no perfect models, the differences in relation to DA stimulation are detectable in all systems, further enhancing the value of the developed pipeline, for new experiments.

  • New Reviewer Comment:  First, see my comment above regarding the p24. The use of primary cell types such as microglia and/or monocyte derived macrophages (MDM) is essential as they represent a more physiological cell type.

Response: As mentioned above, additional graphs were provided, with comments in the text (see line 624 in the discussion).

  • Reviewer’s response:A major concern is the p24 results (Figure S1). On the graph presented, the NT (I assume not treated) showed nearly 1000ng/ml of p24 in the culture supernatant, while the 1 µM DA resulted in ~1200ng/mL, and 10 µM DA resulted in ~ 8000ng/mL.  In all of my years of performing p24 assays, I’ve never had a control of nearly 1000 ng/mL. This experiment shows that the U1 cell line is not latently infected. Finally, why didn’t the authors test DA concentrations of U937 cells?

Response: Corrections have been provided. The model has intrinsic limitations, as do other models. However, the effect of latency reversal agents indicates that despite the relatively high p24 baseline, integrated virus is largely silent in non-treated cells. We compared the levels of p24 with other papers in the literature and found no discrepancy (in pg). Interestingly, latency models in C20 cells also have a higher p24 baseline compared to primary human microglia, which could be attributed to the fact that they are both cell lines. It was interesting, though, to have the models side-by-side as proposed by the reviewer. Regarding U937s, we did test DA in different concentrations in all systems, but we chose one concentration to show results.

Thank you again. For the wonderful exchange, thorough evaluation, and interesting comments. These only make our manuscript more interesting.

Round 2

Reviewer 2 Report (Previous Reviewer 2)

Comments and Suggestions for Authors

In this resubmission of the manuscript by Basova and colleagues, the authors attempt to address the comments from the previous review. This reviewer had four concerns, three of which pertained to whether the U1 cell line was latently infected.  The authors have now corrected the scales on the p24 assays from ng/ mL to pg/mL. However, the results still show that untreated U1 cells express p24 at approximately one ng/mL, which is still indicative that these U1 cells are not latently infected.  The authors should at least perform immunofluorescence assays with an anti-p24 antibody, which would inform readers about the percentage of cells releasing the virus (in this case, p24). Together with a previous study indicating multiple integration sites, and considering that the data from this cell line (a myeloid leukemic cell line) can be related to myeloid cells from HIV-1-positive individuals without leukemia, it suggests that other truly latent primary cell types (several of which have been described in the literature) should be used for these studies.

Author Response

Thank you so much for your suggestion and comments that are all very much valid concerns in relation to the model. We again highlight the adequacy of the model for developing an analysis pipeline and demonstrating effects of a neurotransmitter on the myeloid compartment in a way that is relevant in the context of HIV infection. In round 2 resubmission of the manuscript, we have added experiments indicating that U1 cells have a baseline of under 10% of cells producing p24 protein and that DA significantly increases this amount to about 25% of the cells, with no dose-dependent effect. We chose to demonstrate it using intracellular cytokine staining with p24 antibodies, using flow cytometry methods and analysis, which increase precision of relative number estimates compared to the potentially arbitrary counts using microscopy.

The new experiment can be found in supplementary figure 2. The supplementary figure number adjustments were introduced in the text. We thank the reviewer for the opportunity to provide one more validation experiment. 

This manuscript is a resubmission of an earlier submission. The following is a list of the peer review reports and author responses from that submission.

Round 1

Reviewer 1 Report

Comments and Suggestions for Authors

In this manuscript, Dr. Marcondes and colleagues describe how dopamine, a key neurotransmitter linked to the rewarding effects of psychostimulants in the brain, affects the reactivation of latent HIV using the U1 promonocytic cell line. Briefly, these cells were treated with 10 M dopamine followed by single-cell RNA seq analysis to identify differential gene expression signatures. The data showed that latency was associated with the high expression of histone linkers and components of chromatin organization, together with dysregulation of genes regulating various metabolic pathways and neurodegeneration. In contrast, the addition of dopamine decreased the expression of genes controlling latency while increasing the expression of proinflammatory and pro-HIV genes. The results are interesting as they identify a potential link between psychostimulant drug use and increased HIV transcription in the brain. However, there are some important missing pieces of information that need to be addressed to complete the story.

1. While the activation of HIV gene expression by dopamine is very interesting, the data falls short in demonstrating the successful translation of HIV RNA. The authors should perform a western blot to show successful expression of HIV-specific proteins following activation by dopamine. 

2. Further, the data does not demonstrate the activation and production of infectious viral particles by dopamine. Are the viral particles defective or infectious? Can the virus emerging from dopamine-activated U1 cells infect other target cells (primary or T cell lines)?. These are important questions that should be addressed by the authors to complete the link between psychostimulant use and the production of infectious HIV by latently infected cells in the brain. 

3. No justification is provided for using the U1 cell line. Why not use a latently HIV-infected microglial cell line, as it would have made the findings more impactful and more faithfully reflected the events happening in brain microglia in response to psychostimulant use?

4. All the data provided are at the mRNA levels. In these types of studies, it is customary to confirm the expression of a few select genes using RT-qPCR, as single-cell RNAseq data cannot be considered final. Since the authors have archived brain tissues from SIV infected rhesus macaques treated with methamphetamine, it would be very beneficial to both the authors and readers to confirm protein expression of a few important differentially expressed genes linked to latency reversal and proinflammatory activation by dopamine in the brain. Without this information, the data doesn’t look impactful. 

5. It is not clear why the authors report neutrophil extracellular trap formation as a significantly altered pathway in Figure 4F and what its significance is when the RNA they have processed was derived from the promonocytic U1 cell line. How is it possible to link gene expression signatures identified in U1 cells to the formation of neutrophil extracellular traps?

Minor
The authors should proofread the manuscript carefully to address minor grammatical errors (eg: lines 248-249) in multiple areas.

Author Response

Concerns raised by Reviewer #1

We appreciate the reviewer’s comments to help us identify missing pieces of information.

  1. While the activation of HIV gene expression by dopamine is very interesting, the data falls short in demonstrating the successful translation of HIV RNA. The authors should perform a western blot to show successful expression of HIV-specific proteins following activation by dopamine. 

            Thank you for this comment. We did not show this result in the previous version, because it has been published, and we had included our citations, but we agree that it is important information in the context of the manuscript and as a control of the experiments we are showing. In the revised version of the paper, we included additional supplementary material (with material and methods and a supplementary Figure 1), with results of p24 detection in the supernatant of the DA-stimulated U1 cells, and also in comparison with the maximal latency reversal obtained with the incubation with a latency reversal agent iBet. The figure confirms the dose dependent increment in p24 induced by DA in U1 cells.

  1. Further, the data does not demonstrate the activation and production of infectious viral particles by dopamine. Are the viral particles defective or infectious? Can the virus emerging from dopamine-activated U1 cells infect other target cells (primary or T cell lines)? These are important questions that should be addressed by the authors to complete the link between psychostimulant use and the production of infectious HIV by latently infected cells in the brain. 

            Thank you for this comment and it is an important one. As mentioned above, we have now added levels of p24 of these experiments to supplementary materials. Based on this reviewer’s comment, we have largely expanded the discussion regarding the acknowledged limitations of the model (line 604), providing more references and more information of the virus in U1 cells, but also highlighting a few advantages. In the revised discussion, we better described the defects in Tat causing a partial deficiency that is regarded as a factor in the latency of U1 cells, and also preventing efficient infection. We have stressed that U1 cells can be induced to produce virus, which we now show in the analyzed cultures by p24 ELISA. We have also stressed that this is not a model to study productive infection or viral entry. In fact, we have previously used other models to demonstrate such effects. However, the scope of this present study and the value of U1 cells in that regard lie on the mechanisms that precede infection, productive or not, but are rather related to the host cell regulation of viral gene transcripts expression in the context of a hyperdopaminergic environment, which would otherwise remain silent. We discussed that whether the resulting virus is infectious or not, it does not impact the value of the model for predicting host cell mechanisms of reactivation, and for identifying unique signatures that characterize the phenotypes of latent states. U1 cells as a HIV latent model are useful to our approach above all aiming at developing a method for mechanistic predictions and determination of markers to help identify latent cells in systems where the virus is not tagged by a reporter or within the heterogeneity of target cells in vivo, and to search best strategies to reverse latency by harnessing the immune-dopaminergic response. 

  1. No justification is provided for using the U1 cell line. Why not use a latently HIV-infected microglial cell line, as it would have made the findings more impactful and more faithfully reflected the events happening in brain microglia in response to psychostimulant use?

            Thank you so much for pointing to the lack of a clear rationale for our chosen model and for expressing this concern. The goal of these experiments was to develop a single cell strategy to identify virally infected/latent cells in situations when such cells cannot be sorted or easily identified, or whether they are rare. We have now added this rationale on line 56 of the Introduction. Following this strategy, we are currently performing targeted studies involving the epigenetic mechanisms predicted here, as well as using HIV-infected and latent microglia cells, and validating predicted biomarkers in brain-derived microglia cells in non-human primate models and post-mortem human tissue.

  1. All the data provided are at the mRNA level. In these types of studies, it is customary to confirm the expression of a few select genes using RT-qPCR, as single-cell RNAseq data cannot be considered final. Since the authors have archived brain tissues from SIV infected rhesus macaques treated with methamphetamine, it would be very beneficial to both the authors and readers to confirm protein expression of a few important differentially expressed genes linked to latency reversal and proinflammatory activation by dopamine in the brain. Without this information, the data doesn’t look impactful. 

            We share a similar concern. Indeed, SIV-infected brain tissue is among the next steps in our strategy to identify latent cells in the brain using a panel of signatures. The manuscript presented here, however, is focused on a novel concept of using single cell approaches for “sorting” latent and reactivated cells upon DA exposure, with a focus on systems biology and predictions. The experiments in macaques are currently ongoing and take a long time. Regarding validation, we ahev added some supplementary materials, and we are preparing a manuscript with specific epigenetic mechanisms that derive from the findings of a DA-induced decrease in histone linkers. In the supplementary figure, we added a dose-dependent decrease in H1.5 (as an example), with an inverse correlation with markers of open chromatin such as H3K36me3. These and other related findings will be soon extended and published as a complete story in a separate manuscript focused on this mechanism and its implications to inflammation and viral transcription in the context of substance use, and also in relation to our previous findings on the contribution of Sirtuin-1, which is rigorously a histone linker stabilizer via its deacetylase functions. This is just an example of the value of a work on predictions, which should be made available to all the community regardless of our validation, because other groups may also find genes of interest. It is all very new and exciting!  

  1. It is not clear why the authors report neutrophil extracellular trap formation as a significantly altered pathway in Figure 4F and what its significance is when the RNA they have processed was derived from the promonocytic U1 cell line. How is it possible to link gene expression signatures identified in U1 cells to the formation of neutrophil extracellular traps?

            Yes, this is a fascinating question. Many pathways receive annotation names in gene ontology or Biogrid databases because they have been initially described in other systems, but genes represented in those pathways can be identified in association with any system, including myeloid cells, based on the overrepresentation of matching genes. This is the case here of the pathway neutrophil extracellular trap formation, which happened to be a strong signature and has genes with connections with inflammation, mitochondrial health and integrins. Pathways with apparently unrelated names can potentially show overlap with others that may sound like they make more sense, but we need to report all the findings.

Minor
The authors should proofread the manuscript carefully to address minor grammatical errors (eg: lines 248-249) in multiple areas.

Thank you so much. We found a couple of errors that were fixed. Really appreciate.

Reviewer 2 Report

Comments and Suggestions for Authors

Viral latency generally refers to a state where a virus remains dormant within an infected host cell, neither actively replicating nor causing immediate symptoms, but capable of reactivating and resuming the infectious cycle under specific conditions. In this manuscript, the authors examine the role of dopamine (10µM) on host gene expression and latency reversal in the parental U937 cell line and U1 cells, a derivative of U937 cells with two integrated copies of the NY5 strain of HIV-1.  The single-cell transcriptomics is well described with adequate controls and analyzed with relevant software. However, there are several concerns regarding this study.  The first concern is that the reader is left with numerous either up-regulated or down-regulated genes and no experiments showing they are involved in latency reversal. The second concern is using the U1 cell line, a derivative of the U937 cell line first isolated from an individual with adult acute monocytic leukemia. In a previous study, investigators investigated the integration sites in several “latently infected” cell lines, including the U1 cell line. They showed that HIV-1 was mainly integrated into chromosome 2 and the X chromosome, with less frequent integration into chromosome 19. Based on sequence analysis, these investigators found a total of 28 unique HIV integration sites, indicating that these cell lines are not clonal (Symons et al., Retrovirology, 14:2, 2017). The above study suggests that the results of the transcriptomics studies could vary based on the number of cell populations with different HIV-1 integration sites. The third concern is whether the data from these transcriptional studies can be related to myeloid cells from HIV-1-positive individuals without leukemia. Better models have been described for assessing HIV-1 latency in primary monocyte-derived macrophages (MDM) (Wong et al., J. Virology; 95:(19):e0022721, 2021).  A fourth concern relates to the expression of HIV-1. The U1 cell line has two integrated proviruses, both of which have mutations in tat (in one integrant the tat initiation codon is mutated to a leucine resulting in no Tat produced; in the second integrant, there is an amino acid substitution (H to L) at position 13, resulting in significantly decreased transactivation activity and virus production (see Emiliani et al., J. Virology, 72:1666-1670,1998). While the authors show HIV-1 transcripts in their studies, this should be confirmed with studies measuring the levels of viral proteins released (either by p24 or reverse transcriptase assays) following “latency reversal” by dopamine. Such studies need to be included to support their data. Finally, the manuscript would be strengthened by a) assessing transcripts in the U1 cell line treated with phorbol 12-myristate 13-acetate (PMA) or other known latency reversal agents and comparing them with those of the dopamine studies; and b) assessing transcripts in the U1 cell line treated with a dopamine antagonist before dopamine treatment.