# Supplementary material for: A Single-Cell Perspective on the Effects of Dopamine in the Regulation of HIV Latency Phenotypes in a Myeloid Cell Model
Source: Viruses. 2025 Jun 25;17(7):895. doi: 10.3390/v17070895 (PMC12299422; doi:10.3390/v17070895)
Supplement: Supplementary file 1 [file viruses-17-00895-s001.zip › viruses-3663317-supplementary.pdf]

Supplementary Material and Methods and Figures

**P24 ELISA in U1 culture supernatants** – Supernatant from stimulated cells was collected at the same time as cells were processed for single cell approaches and were stored at -20 °C until used in assays. Positive controls for p24 maximum production were U1 cells stimulated with 1uM of the latency reversal agent bromodomain inhibitor iBet151 (Cayman Chemicals, Ann Arbor, MI), and negative controls contained DMSO at molarity dilution. Levels of p24 antigen in U1 and U1+iBet were determined by ELISA using HIV-1 p24 ELISA assay kit (Xpress Bio, Frederick, MD), according to the manufacturer's instructions.

**P24 Intracellular staining** - Following treatment with DA performed as described, or with the latency reversal agent phorbol-myristate-acetate (2mM, PMA) as a positive control, the cells were washed with PBS through centrifugation, and surface staining with anti-RAGE (Novus) monoclonal was performed in staining buffer (PBS with 2% FBS and 0.2% NaN3). Following that, the cells were fixed and permeabilized with cold Perm Buffer III (BD Bioscience) on ice for 30 minutes. Cells were washed and stained using monoclonal anti-p24 FITC (clone KC57) from Beckman Coulter antibodies for 1 hour at room temperature protected from light. The cells were washed with staining buffer and measured by flow cytometry immediately. Acquisition was performed in a Cytoflex flow cytometer (Beckman Coulter). Analysis was performed in FlowJo software (BD Bioscience).

**Nuclear fraction extraction and Western blots -** The cells treated as described in Material and Methods were centrifuged at 2,500 g for 5 min, and the pellets were washed twice in PBS, and in 0.5% Triton X buffer, for isolation of nuclear fraction using EpiQuick Nuclear Extraction kit (OP-0002, Epigentek), before being resuspended in RIPA buffer (Thermofisher), added 1 mini tablet Complete (Roche protease inhibitors) for protein extraction. Protein concentration was measured using Bicinchoninic Acid (BCA) protein assay kit (23227, Thermo), and proteins were stored in -20$^{o}$C until use. Western blots for were performed by loading 50 µg of nuclear lysates in 4x Laemli sample buffer denatured for 5 minutes at 95oC and then resolved by SDS-PAGE electrophoresis in 4-20% gradient gels (Biorad). Molecular weight was monitored using 5 µl of PageRuler Plus Prestained Protein Ladder (Thermofisher) in parallel wells. Proteins were transferred to a PVDF membranes overnight. The membranes were blocked with 5% non fat dried milk in TBST (Tris-buffered saline containing 0.1% Tween20) for 1 h at room temperature. Immunoblotting was carried out using the following antibodies: Histone 1.5 phospho serine 17 was detected using a rabbit polyclonal AB_2793508 (Active Motif) at a dilution of 1:1,000; total H1 was detected using AB_2636962 (Active Motif) at 1:1,000; H3K36me3 was detected using AB_2615073 at 1:1,000. Loading control was performed by the detection of GAPDH using rabbit polyclonal NB100-56875SS (Proteintech) at 1:2,000 dilution. All antibodies were incubated overnight at 4$^{o}$C on a rocking platform, followed by a Goat anti-Rabbit IgG (H+L) HRP conjugate (Cell Signaling) for 1 h at room temperature. Chemiluminescent detection was performed using SuperSignal Pico PLUS substrate (Thermofisher) using the ChemiDoc system (Biorad). Densitometry analysis and quantification was performed in ImageJ Fiji (NIH), using the band of interest normalized to each respective internal GAPDH loading control.

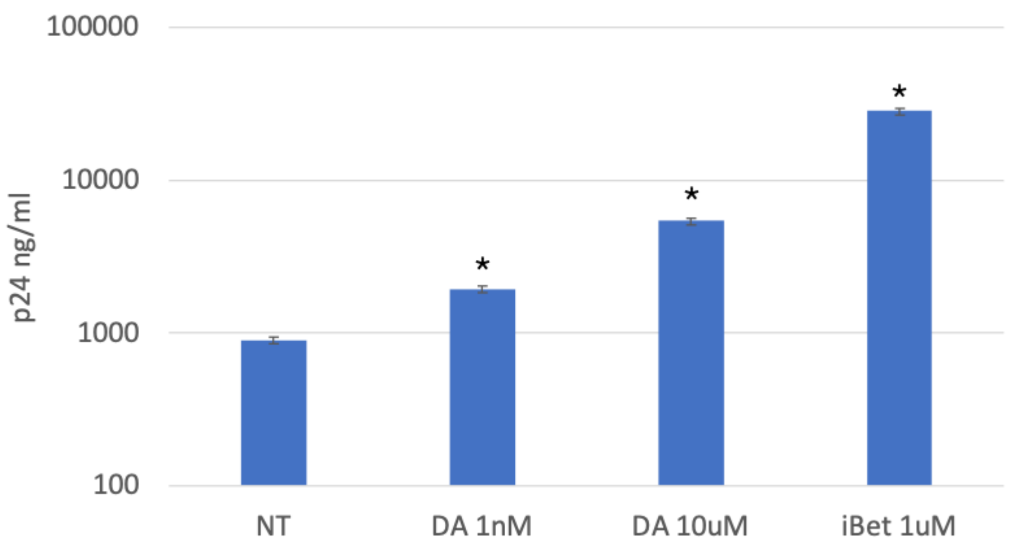


Supplementary Figure S1 – Detection of p24 in the supernatant of U1 cells stimulated with DA a latency reversal agent. P24 was measure by ELISA, in supernatants at 24 hrs after stimulation with 1 or 10uM of DA, or 1uM of the latency reversal agent iBet at 1uM. *p<0.05 in comparison with non-treated vehicle conditions.

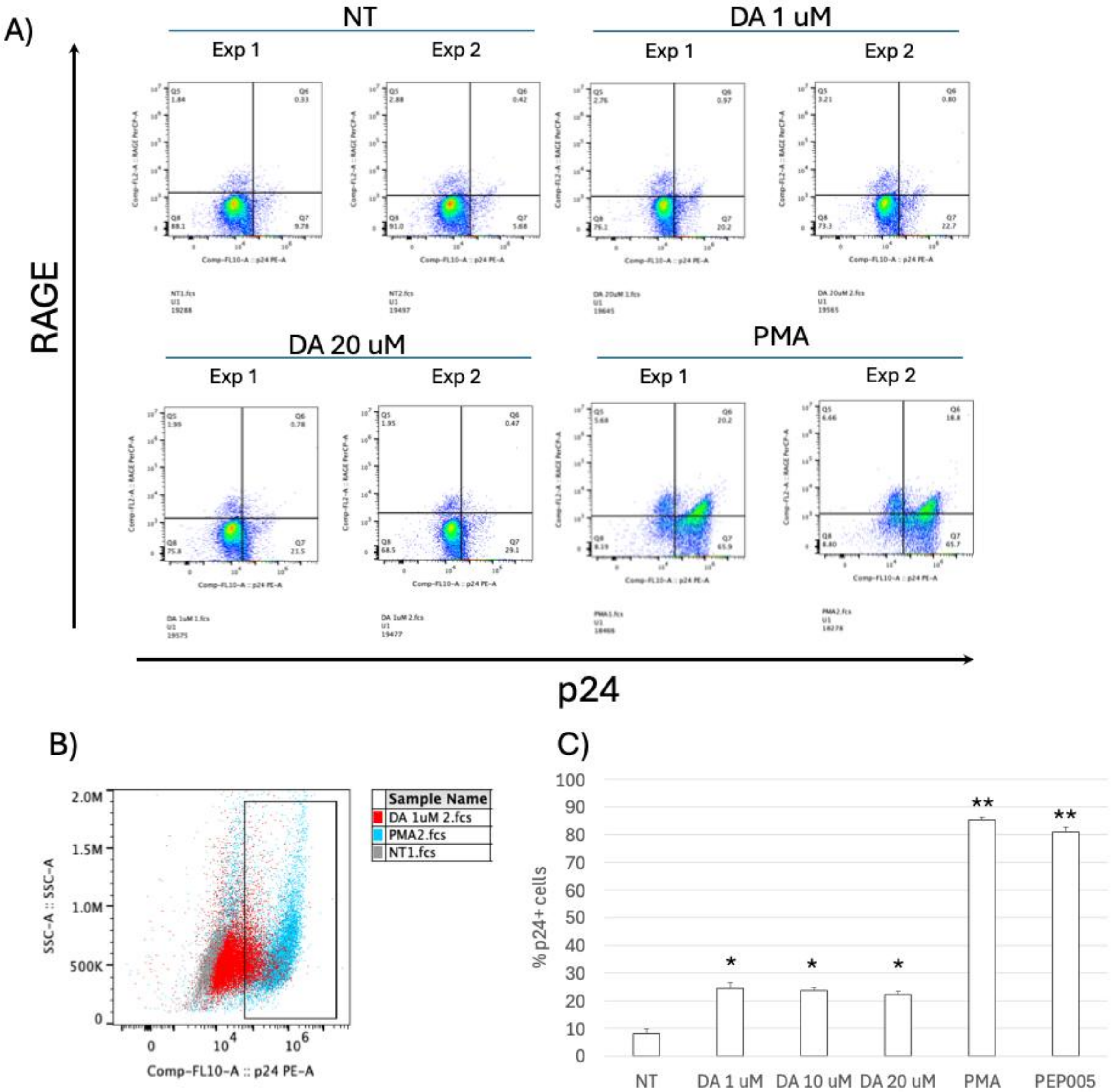


Supplementary Figure S2 – Detection of p24+ cells by flow cytometry. U1 cells were stimulated with DA at 1 and 20 uM, or two latency reversal agents, PMA at 5 ng/ml or PEP005 at 10nM for 24hrs. Following staining with a surface activation marker (RAGE), intracellular p24 was detected with a specific antibody for estimating the relative number of cells producing viral proteins. A) Flow cytometry plots of two independent experiments showing non-treated (NT) cells, cells treated with 1 and 20 uM of DA, and the latency reversal agent phorbol-myristate-acetate (PMA). B) Overlap plot of p24 fluorescence intensity in NT (gray), PMA (blue) and DA 1uM (red). C) Percentage of cells positive for p24 in different conditions. *p<0.05, **p<0.001.

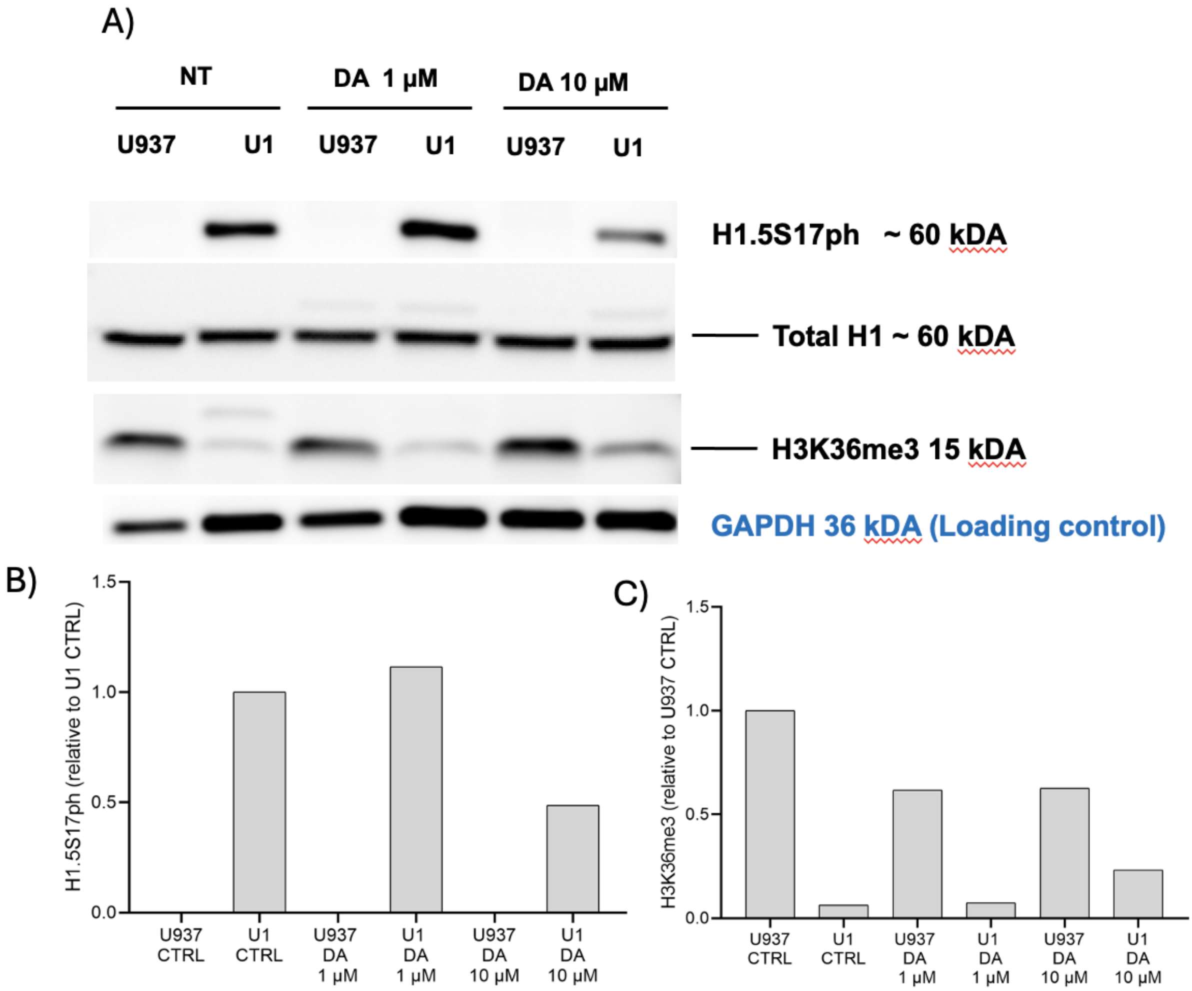


Supplementary Figure S3 – Protein validation of changes caused by DA in U1 cells. A) Representative western blot image of the detection Histone linker H1.5 pS17, in respect to total H1 and H3K36me3 open chromatin marker in U937 and U1 cells, following exposure to DA 1 and 10uM for 24 hrs. B) Fold change of H1.5 pS17 in relation to U1 unstimulated control in quantified and GAPDH normalized bands. C) Fold change of H3K36me3 in relation to U937 unstimulated control in quantified and GAPDH normalized bands.

Supplementary Table S1 – Individual cluster's characteristics used for grouping according to response patterns, their predicted regulators, and significant signatures aligned with HIV-linked gene enrichment patterns identified in Gene Set Enrichment Analysis GSEA database (M40872, M41082).

| **Cluster #** | **Predominant Pattern** | **Predicted upstream activation Regulators** | **HIV-linked Molecular Signatures from GSEA database** |
|---|---|---|---|
| **1** | U937 stimulated with DA (Control+DA) | PTH<br>FAM20A<br>STAT4 | 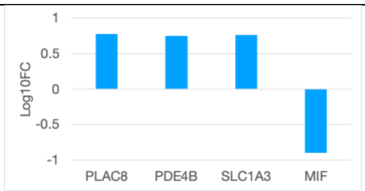 |
| **2** | Unstimulated U1 (Latency+Vehicle) | EIF5<br>MTREX | HIV<br>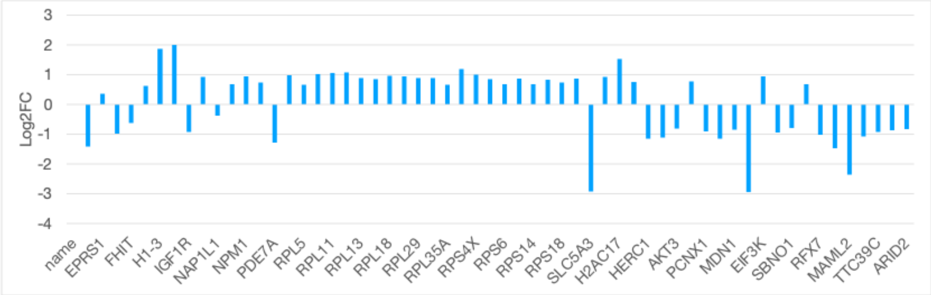 |
| **3** | U1 treated with DA (Latency + DA) | LMNB1<br>TP53<br>SIRT1 | 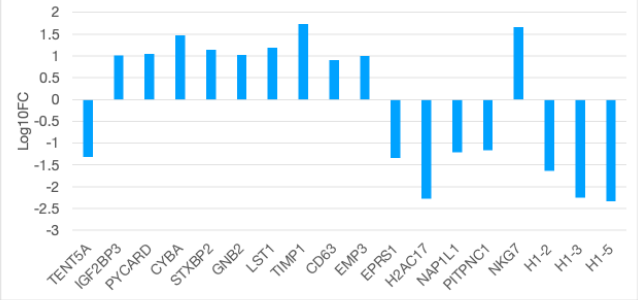 |
| **4** | Untreated U937 (Control+Vehicle) | ADAM10<br>FAAH | 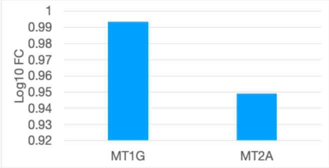 |
| **5** | U937 and U1 stimulated with DA (DA) | - | No significant genes linked to HIV studies. |

| | | | |
|---|---|---|---|
| **6** | U1 stimulated with DA (Latency+DA) | GH1<br>TNF | 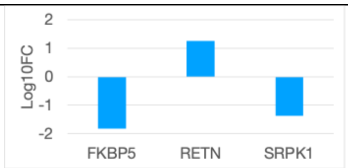 |
| **7** | Untreated U1 (Latency+Vehicle) and U937 stimulated with DA | CDKN1B | 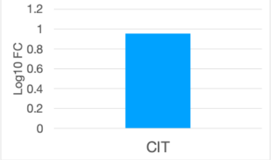 |
| **8** | U937 stimulated with DA (DA) | IL4<br>TNF | 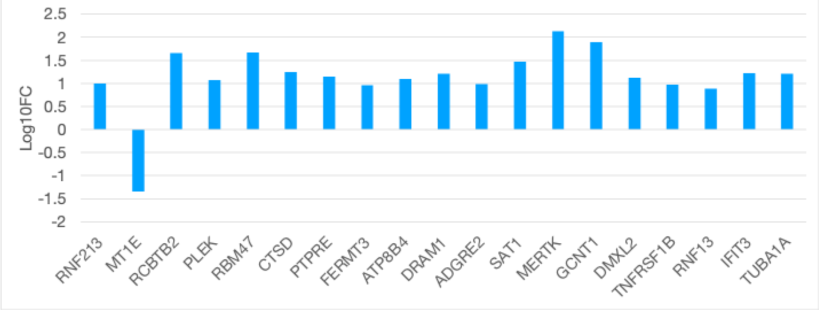 |
| **9** | Untreated U937 (Control+Vehicle) | ADAM10<br>FAAH | 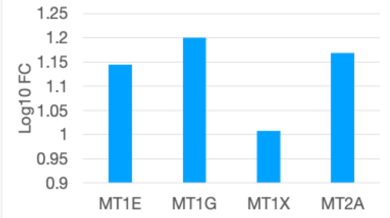 |
| **10** | Untreated U1 (Latency+Vehicle) | CSF3<br>FAM20A<br>STAT4 | 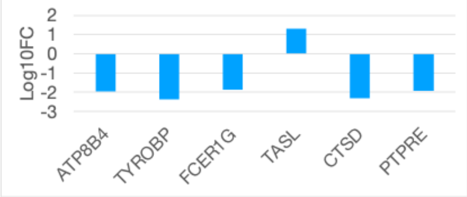 |
| **11** | Untreated U937 (Control+ Vehicle) | CDKN1B | 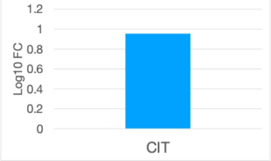 |
| **12** | U1 stimulated with DA (Latency +DA) | | No significant genes |
| **13** | U937 stimulated with DA (Control + Vehicle) | ATF6<br>IL6<br>FAM20A<br>FAM20C | 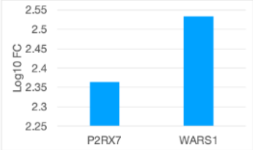 |
| **14** | All conditions | | |
| **15** | All conditions | | |

Supplementary Table S2 – Fifty significantly most upregulated genes in correlation with positive transcription of HIV-1 Gag-Pol in U1 cells following DA.

| Feature Name | Gag-Pol-pos Average | Gag-Pol-pos Log2 Fold Change | Gag-Pol-pos P-Value | Feature ID | Gene Name |
|---|---|---|---|---|---|
| gag-pol | 1.92925241 | 15.7790888 | 1.42955e-318 | gene0 | gag-pol |
| vif | 0.07761574 | 3.72899581 | 2.97E-25 | gene2 | vif |
| gag | 0.01039828 | 3.6839694 | 7.45E-06 | gene1 | gag |
| tat | 0.08987085 | 3.48136588 | 1.41E-31 | gene4 | tat |
| env | 0.77615737 | 3.38020176 | 6.89E-43 | gene7 | env |
| vpu | 0.02023951 | 3.24012168 | 1.11E-21 | gene6 | vpu |
| vpr | 0.01485469 | 3.10346655 | 4.22E-08 | gene3 | vpr |
| nef | 0.15226054 | 2.9473553 | 7.77E-24 | Gene8 | |
| ADAMDEC1 | 0.00259957 | 2.88350087 | 0.00229883 | ENSG00000134028 | ADAM like decysin 1(ADAMDEC1) |
| PRR33 | 0.00241389 | 2.24339681 | 0.03831853 | ENSG00000283787 | proline rich 33(PRR33) |
| CYP8B1 | 0.0022282 | 2.22959101 | 0.05392663 | ENSG00000180432 | cytochrome P450 family 8 subfamily B member 1(CYP8B1) |
| EDN1 | 0.00742734 | 2.20863139 | 0.00394047 | ENSG00000078401 | endothelin 1(EDN1) |
| rev | 0.00241389 | 2.15593397 | 0.04901094 | gene5 | |
| CALHM3 | 0.00389936 | 2.08811859 | 0.00767585 | ENSG00000183128 | calcium homeostasis modulator 3(CALHM3) |
| nef | 0.01893973 | 2.02451033 | 1.44E-08 | gene9 | |
| EMP1 | 0.04085039 | 1.91106149 | 9.31E-10 | ENSG00000134531 | epithelial membrane protein 1(EMP1) |
| GJA1 | 0.00297094 | 1.87964854 | 0.05643691 | ENSG00000152661 | gap junction protein alpha 1(GJA1) |
| AHNAK2 | 0.01281217 | 1.87882438 | 2.42E-05 | ENSG00000185567 | AHNAK nucleoprotein 2(AHNAK2) |
| OR5B21 | 0.00371367 | 1.874163 | 0.03390157 | ENSG00000198283 | olfactory receptor family 5 subfamily B member 21(OR5B21) |
| RGCC | 0.00557051 | 1.86667624 | 0.00357954 | ENSG00000102760 | regulator of cell cycle(RGCC) |
| KL | 0.00742734 | 1.79359389 | 0.04696362 | ENSG00000133116 | klotho(KL) |
| GBP2 | 0.01169807 | 1.77783041 | 0.00489234 | ENSG00000162645 | guanylate binding protein 2(GBP2) |
| CD109 | 0.02933801 | 1.74892485 | 0.0015821 | ENSG00000156535 | CD109 molecule(CD109) |
| VWA5B1 | 0.00464209 | 1.71021685 | 0.02634348 | ENSG00000158816 | von Willebrand factor A domain containing 5B1(VWA5B1) |
| CD86 | 0.02432455 | 1.70564895 | 2.50E-06 | ENSG00000114013 | CD86 molecule(CD86) |
| IL7R | 0.0113267 | 1.68979848 | 0.00040197 | ENSG00000168685 | interleukin 7 receptor(IL7R) |
| SPP1 | 0.05180572 | 1.68880796 | 8.61E-05 | ENSG00000118785 | secreted phosphoprotein 1/ Osteopontin (SPP1) |
| SLCO2B1 | 0.02525297 | 1.67609298 | 1.81E-06 | ENSG00000137491 | solute carrier organic anion transporter family member 2B1(SLCO2B1) |
| HMOX1 | 0.23767498 | 1.61635137 | 1.89E-08 | ENSG00000100292 | heme oxygenase 1(HMOX1) |
| MAFB | 0.30099309 | 1.60975203 | 8.15E-09 | ENSG00000204103 | MAF bZIP transcription factor B(MAFB) |
| SLC6A12 | 0.00649893 | 1.60596689 | 0.01088816 | ENSG00000111181 | solute carrier family 6 member 12(SLC6A12) |

| | | | | | |
|---|---|---|---|---|---|
| IL1B | 0.04493543 | 1.60263118 | 6.97E-05 | ENSG00000125538 | interleukin 1 beta(IL1B) |
| KLHL4 | 0.00538482 | 1.56157277 | 0.03390157 | ENSG00000102271 | kelch like family member 4(KLHL4) |
| LILRA6 | 0.01392627 | 1.55468639 | 0.00039153 | ENSG00000244482 | leukocyte immunoglobulin like receptor A6(LILRA6) |
| CLEC7A | 0.02135361 | 1.50612033 | 8.61E-05 | ENSG00000172243 | C-type lectin domain containing 7A(CLEC7A) |
| C5AR1 | 0.27016961 | 1.47470351 | 3.40E-07 | ENSG00000197405 | complement C5a receptor 1(C5AR1) |
| ITGB5 | 0.0259957 | 1.45665217 | 0.00031986 | ENSG00000082781 | integrin subunit beta 5(ITGB5) |
| FFAR2 | 0.10156892 | 1.44926559 | 2.12E-06 | ENSG00000126262 | free fatty acid receptor 2(FFAR2) |
| PTPRO | 0.02896664 | 1.42688189 | 9.60E-05 | ENSG00000151490 | protein tyrosine phosphatase receptor type O(PTPRO) |
| AKR1C8P | 0.00742734 | 1.41855446 | 0.02424888 | ENSG00000264006 | Aldo-Keto Reductase Family 1 Member C8 |
| TXLNB | 0.01429764 | 1.41766336 | 0.00143092 | ENSG00000164440 | taxilin beta(TXLNB) |
| ITGB7 | 0.01485469 | 1.40956968 | 0.04687077 | ENSG00000139626 | integrin subunit beta 7(ITGB7) |
| ADORA2B | 0.01225512 | 1.39360662 | 0.02474649 | ENSG00000170425 | adenosine A2b receptor(ADORA2B) |
| AQP8 | 0.01318353 | 1.38679837 | 0.01860222 | ENSG00000103375 | aquaporin 8(AQP8) |
| SLC4A9 | 0.01299785 | 1.38611666 | 0.00575233 | ENSG00000113073 | solute carrier family 4 member 9(SLC4A9) |
| MTUS1 | 0.01838268 | 1.31171376 | 0.01512948 | ENSG00000129422 | microtubule associated scaffold protein 1(MTUS1) |
| TRIB1 | 0.02766685 | 1.28982115 | 0.00075654 | ENSG00000173334 | tribbles pseudokinase 1(TRIB1) |
| CD36 | 1.10667414 | 1.28847462 | 1.75E-05 | ENSG00000135218 | CD36 molecule (CD36 blood group)(CD36) |
| CCL24 | 0.05477666 | 1.2857457 | 0.00017591 | ENSG00000106178 | C-C motif chemokine ligand 24(CCL24) |
| C5AR2 | 0.05533371 | 1.28500413 | 0.0002038 | ENSG00000134830 | complement C5a receptor 2(C5AR2) |

Supplementary Table S3 – Genes significantly downregulated in correlation with positive transcription of HIV-1 Gag-Pol in U1 cells following DA.

| Feature Name | Gag-Pol-pos Average | Gag-Pol-pos Log2 Fold Change | Gag-Pol-pos P-Value | Feature ID | Gene Name |
|---|---|---|---|---|---|
| VCX3B | 0.00055705 | -2.3321424 | 0.03262046 | ENSG00000205642 | variable charge X-linked 3B(VCX3B) |
| MYL10 | 0.00445641 | -1.9969175 | 0.00014469 | ENSG00000106436 | myosin light chain 10(MYL10) |
| HLA-DRA | 0.0259957 | -1.8469972 | 7.45E-06 | ENSG00000204287 | major histocompatibility complex, class II, DR alpha(HLA-DRA) |
| KANK4 | 0.00854145 | -1.6800255 | 0.00053318 | ENSG00000132854 | KN motif and ankyrin repeat domains 4(KANK4) |
| VCX | 0.00352799 | -1.5684595 | 0.02107096 | ENSG00000182583 | variable charge X-linked(VCX) |
| PRTN3 | 3.57867965 | -1.5236588 | 7.22E-05 | ENSG00000196415 | proteinase 3(PRTN3) |
| NRXN3 | 0.13499196 | -1.4204349 | 0.00075654 | ENSG00000021645 | neurexin 3(NRXN3) |
| CACHD1 | 0.01689721 | -1.3200067 | 0.00803994 | ENSG00000158966 | cache domain containing 1(CACHD1) |
| HLA-DRB5 | 0.01188375 | -1.1998018 | 0.03584391 | ENSG00000198502 | major histocompatibility complex, class II, DR beta 5(HLA-DRB5) |

| | | | | | |
|---|---|---|---|---|---|
| ELANE | 0.91839099 | -1.1571599 | 0.00682501 | ENSG00000197561 | elastase, neutrophil expressed (ELANE) |
| H1-4 | 1.07826455 | -1.1308403 | 0.00987475 | ENSG00000168298 | H1.4 linker histone, cluster member(H1-4) |
| H1-5 | 0.42651519 | -1.1273627 | 0.01078151 | ENSG00000184357 | H1.5 linker histone, cluster member(H1-5) |
| FGF13 | 0.09432726 | -1.0647902 | 0.03586766 | ENSG00000129682 | fibroblast growth factor 13(FGF13) |
| HLA-DRB1 | 0.04827773 | -1.0527371 | 0.04244114 | ENSG00000196126 | major histocompatibility complex, class II, DR beta 1(HLA-DRB1) |
| XAGE1B | 0.0531055 | -1.0311376 | 0.04913261 | ENSG00000204382 | X antigen family member 1B(XAGE1B) |
